# Topical Formulation Comprising Fatty Acid Extract from Cod Liver Oil: Development, Evaluation and Stability Studies

**DOI:** 10.3390/md14060105

**Published:** 2016-06-01

**Authors:** Biljana Ilievska, Thorsteinn Loftsson, Martha Asdis Hjalmarsdottir, Gudrun Marta Asgrimsdottir

**Affiliations:** 1 Faculty of Pharmaceutical Sciences, University of Iceland, Hofsvallagata 53, 107 Reykjavik, Iceland; thorstlo@hi.is; 2 Faculty of Medicine, University of Iceland, Sæmundargata 2, 101 Reykjavik, Iceland; hjalmars@hi.is; 3 Lipid Pharmaceuticals, Grandagardur 16, 101 Reykjavik, Iceland; gudrun@lipid.is

**Keywords:** PUFA (polyunsaturated fatty acid), DHA (docosahexaenoic acid), EPA (eicosapentaenoic acid), FA (fatty acid), ointment

## Abstract

The purpose of this study was to develop a pharmaceutical formulation containing fatty acid extract rich in free omega-3 fatty acids such as eicosapentaenoic acid and docosahexaenoic acid for topical use. Although the health benefits of cod liver oil and other fish oils taken orally as a dietary supplement have been acknowledged and exploited, it is clear that their use can be extended further to cover their antibacterial properties. *In vitro* evaluation showed that 20% (*v*/*v*) fatty acid extract exhibits good activity against strains of the Gram-positive bacteria *Staphylococcus aureus*, *Enterococcus faecalis*, *Streptoccoccus* *pyogenes* and *Streptoccoccus* *pneumonia*. Therefore, free polyunsaturated fatty acids from cod liver oil or other fish oils can be used as safe and natural antibacterial agents. In this study, ointment compositions containing free fatty acids as active antibacterial agents were prepared by using various natural waxes and characterized. The effects of different waxes, such as carnauba wax, ozokerite wax, laurel wax, beeswax, rice bran wax, candelilla wax and microcrystalline wax, in the concentration range of 1% to 5% (*w*/*w*) on the ointment texture, consistency and stability were evaluated. The results showed significant variations in texture, sensory and rheological profiles. This was attributed to the wax’s nature and chain composition. Microcrystalline wax gave the best results but laurel wax, beeswax and rice bran wax exhibited excellent texturing, similar sensory profiles and well-balanced rheological properties.

## 1. Introduction

The nutritional and medical benefits of fish oils that are rich in polyunsaturated fatty acids (PUFAs) have been extensively investigated. For example, numerous studies have confirmed that PUFAs possess anti-inflammatory properties and, thus, regular fish oil consumption is thought to reduce the risk of cardiovascular diseases [1,2]. Fish liver oil for human consumption is commonly extracted from Atlantic cod (*Gadus morhua*). The fatty acid composition of cod liver oil is shown in Table 1. It contains saturated, monounsaturated and various PUFAs, including both eicosapentaenoic acid (EPA) and docosahexaenoic acid (DHA) [3]. Antibacterial, antiviral and antifungal effects have also been associated with PUFAs [4,5,6,7,8]. It has been reported that the unsaturated fatty acids’ (FAs) potency increases with the degree of unsaturation, while esterified FAs are less effective than free FAs [9]. Similarly, for cod liver oil, it has been shown that the extract of free FAs from cod liver oil is more potent than cod liver oil containing the same FAs in the form of triglycerides [10,11]. Besides the strong antibacterial properties, free FAs also possess antiviral properties and the FAs extract from cod liver oil has been shown to cause a significant reduction of herpes simplex virus type 1 HSV-1 activity. 1% FAs extract caused a 50,000 fold or greater (≥4.7 log10) reduction of viral infectivity in 10 min [12]. In addition, PUFAs have been used as penetration enhancers in transdermal and buccal drug formulations [13]. To date, the mechanisms by which FAs act as antibacterial agents have not been resolved, and this continues to be a subject of some research effort. Although the exact mode of the bactericidal action of fatty acids still remains unclear, the cellular membrane is thought to be the primary target [14,15].

Ointments are semi-solid, homogenous, viscous pharmaceutical preparations usually intended for external use such as on skin or mucosal membranes [16]. Waxes are frequently used as hydrophobic excipients in topical drug formulations to obtain desired viscosity and consistence [17]. Waxes are a large and complex group of lipophilic compounds that generally consist of mixtures of esters of fatty acids with long-chain monohydric alcohols [18]. Today, a wide variety of natural and synthetic waxes are utilized in cosmetics and pharmaceutical products. Seen from a pharmacological aspect, waxes are recognized as a potential excipient or enhancing the aesthetic and maximizing the therapeutic benefits of topical formulations by increasing the viscosity and prolonging the drug retention on the skin surface [19,20]. The objective of this study is to evaluate the antibacterial properties of the fatty acid extract from cod liver oil and design a suitable pharmaceutical formulation for topical administration of the fatty acid extract. This article briefly describes the use of waxes as lipophilic excipients for ointments and how selected waxes influence the ointments’ rheological and sensory properties.

## 2. Results

The compositions of the ointments tested are shown in Table 2. The final ointment formulation was obtained by adding 40% (*w*/*w*) fatty acid extract to a mixture of Vaseline, coconut butter, wax base, lavender oil and lemon oil.

Compositions of ointments tested: wax is carnauba (C1, C2, C3), ozokerite (O1, O2, O3), laurel (L1, L2, L3), bees (B1, B2, B3), candelilla (Cd1, Cd2, Cd3), rice bran (Rb1, Rb2, Rb3) and microcrystalline (M1, M2, M3). Each wax was tested at three levels (1%, 3% and 5% (*w*/*w*)).

### 2.1. *In Vitro* Evaluation of Antibacterial Activity of Fatty Acid Extract

The fatty acid extract from fish liver oil was subjected to antibacterial efficiency testing using strains of representative species of both Gram-positive and Gram-negative bacteria. As determined by the agar dilution method, the extract showed antibacterial activity against all four tested Gram-positive strains: *Staphylococcus aureus*, *Enterococcus faecalis*, *Streptococcus pyogenes* and *Streptococcus pneumonia*. However, the extract did not show antibacterial activity against the Gram-negative bacteria *Pseudomonas aeruginosa* and *Escherichia coli* in this study (MIC > 16.384 μg/mL) (Table 3).

### 2.2. Evaluation of Ointments

The ointments were evaluated for their physical properties such as their spreadability, extrudability and pH as well as their appearance. Spreadability, extrudability and pH studies were carried out in triplicate and average values are reported.

#### 2.2.1. Spreadability Measurements

Ointment spreadability was found to be inversely proportional to the wax concentration. As the wax amount was increased the ointments became more viscous and, consequently, increasing the wax concentration from 1% to 5% led to decreased spreadability. Spreadability measurements are presented in Table 4. Formulations B1, B2, and B3 exhibited the highest spreadability while formulations C1, C2, C3 and O1, O2 and O3 displayed the lowest spreadability. No statistically significant difference was found between formulations L1, L2, L3 and M1, M2, M3. Formulations Cd1, Cd2, Cd3 and Rb1, Rb2, Rb3 showed good spreadability and were not as fluid as B1, B2, B3 and not as stiff as C2, C3, O2, O3.

The spreading values of these formulations were in the following order: B1 > B2 > B3 > L1 > M1 > L2 > Rb1 > M2 > C1 > O1 > L3 > Cd1 > Rb2 > M3 > Cd3 > Cd2 > Rb3 > O2 > C2 > C3 > O3.

#### 2.2.2. Extrudability Measurements

Increasing the wax concentration resulted in decreased extrudability. In nearly all cases the increased wax concentration from 1% to 5% led to a considerable decrease in tube extrudability except in formulations Cd1, Cd2, Cd3, and M1, M2, M3, and L2, L3 wherein the decrease in extrudability with the increasing wax concentration was less pronounced. Increasing the concentration of rice bran wax and microcrystalline wax had a less dramatic but still pronounced effect on extrudability. As can be seen in Table 4, ointments formulated with carnauba wax had the lowest extrudability, while ointments formulated with beeswax were less viscous and had the highest extrudability.

The extrudability values of these formulations were in the following order: Rb3 > C3 = Rb2 > O3 > C2 > L3 > L2 > L1 > O2 > M3 > Cd3 > Rb1 > O1 = C1 > M1 = Cd1 > B3 > Rb3 > M2 > B2 > B1.

#### 2.2.3. Determination of pH

As shown in Table 4, the pH of the ointments ranged from 3.83 to 5.44, and, in general, increasing the wax concentration from 1% to 5% resulted in a slight pH decrease with the exception of L1, L2, and L3 wherein the pH slightly increased. In fact, pH increased when the laurel wax concentration was increased from 1% to 3%, but almost no changes were found when the laurel wax concentration was increased from 3% to 5%. The pHs of L1 (3.83 ± 0.03), B3 (3.96 ± 0.01), Cd2 (3.84 ± 0.02), Cd3 (3.83 ± 0.02) were slightly below 4. Of all the ointments tested, the lowest pH was observed in L1 (contains 1% laurel wax), and the highest in C1 (contains 1% carnauba wax).

#### 2.2.4. Determination of Lipid Oxidation

**Figure 1:**
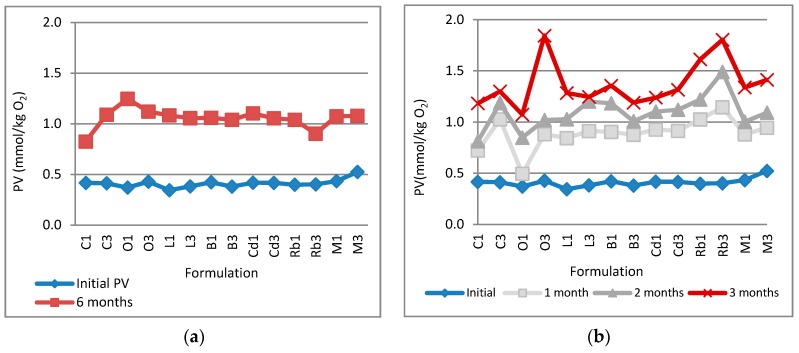
Lipid oxidation measured as peroxide value: (**a**) real-time tested period; (**b**) accelerated tested period.

The results of the peroxide value (PV) assay (Figure 1) represent how stable the PUFAs were during storage for six months at ambient temperature (*i.e.*, 22–23 °C) and for three months under accelerated conditions (*i.e.*, 40 °C). The content of the primary lipid oxidation products measured as the PV of the extract was in the range 1.073–1.804 mmol/kg O_2_ at the end of three months of accelerated testing and 0.824–1.298 mmol/kg O_2_ at the end of six months of storage at room temperature. In both cases no statistically significant differences in the PV values were observed when the wax content was increased.

#### 2.2.5. Sensory Evaluation

The physical appearance of the ointments was light brown, the color they got from the extract present in high quantity (40% *w*/*w*) in all the ointments tested. Regardless of the type of wax used and its concentration, only minor changes in the brown color were observed. All formulations had a strong distinctive smell of fish odor. The overall best results were obtained when beeswax, rice bran wax, laurel wax or microcrystalline wax were used in the ointment base. Overall cosmetic acceptability was rated satisfactory for C1, C2, and Cd1 and Cd2. Specifically, formulations containing beeswax as well as laurel wax had an advantage of providing an additional glossy appearance without having a negative impact on the homogeneity and greasiness. Compared to other unsatisfactory formulations that had a rough, gritty texture such as ointments containing carnauba wax or ozokerite wax, ointments that contain candelilla wax differ by possessing large visible particles distributed throughout a glossy, homogenous ointment base.

## 3. Discussion

### 3.1. Antibacterial Activity of the Fatty Acid Extract

In the present study, the antibacterial effect of the fatty acid extract obtained by the hydrolysis of cod liver oil was investigated and ointments containing the extract were prepared. The fatty acid extract showed antibacterial activity against all the strains of the Gram-positive bacteria tested and their MICs were similar, 128–512 μg/mL. The fatty acid extract did not show antibacterial activity against the Gram-negative strains in the highest concentration measured. These results agree with previous studies where PUFAs were shown to be more potent against Gram-positive bacteria than Gram-negative bacteria [6,9,21]. A possible explanation for the difference in susceptibility lies in the difference in the cell wall structure of Gram-positive and Gram-negative bacteria [14,22]. Some accumulation of the fatty acid extract was observed on the agar surface but it gradually decreased upon progressive dilution to 128 μg/mL. The agar dilution method is probably not applicable for antibacterial measurements of free FAs at high concentrations due to the accumulation of the fatty acid extract on the agar surface.

### 3.2. Ointment Design and Evaluation

During pharmaceutical formulation, selection of appropriate excipients and their amount is critical and various factors have to be considered such as the formulation appearance, odor, color and firmness, all of which can influence the patient acceptability. Other important factors include the chemical stability of the active pharmaceutical ingredient (*i.e.*, the drug), and the overall chemical and physical stability of the formulation. Sometimes, it is necessary to use several different excipients to obtain the vehicle of desired physicochemical properties. The main reason is that it is frequently impossible for a single excipient to produce an ideal vehicle (here an ointment base), but a combination of several excipients frequently will give vehicles with enhanced aesthetics as well as increase drug potency and bioavailability [23,24]. Vaseline is the most common lipophilic excipient in dermal formulations and its usage is sometimes associated with its occlusive properties that can enhance penetration of the active ingredient into skin and improve the therapeutic efficacy of the product [17]. Our experience is, however, that the use of Vaseline alone as an excipient where prolonged drug retention is desirable is not likely to result in acceptable formulation. Waxes were chosen as viscosity-building excipients to improve the ointments’ performance and give them optimal rheological profiles. The combination of Vaseline and waxes produced pharmaceutically acceptable ointments that were less greasy than pure Vaseline and that were retained on the skin for a sufficiently long time to allow for extended drug delivery. The spreadability and extrudability profiles were found to be satisfactory for a topical dosage form. Spreadability and tube extrudability do, in principle, correlate with the rheological properties and present a simple and accurate approach to understanding the relationship between viscosity and the force needed to squeeze the ointment out of a tube [25,26,27]. Both measurements showed the ointment’s tendency to become stiffer upon incorporation of wax. The rheological effect can be explained by the nature of the fatty acid moieties of the waxes. The greater the saturated fatty acid fraction of the wax structure, the more viscous the ointments will be, and, conversely, the viscosity of the ointments will decrease with increasing content of unsaturated fatty acids [28]. Spreadability and extrudability values were influenced by both the amount and type of wax used.

In most cases the pH of the ointments was within acceptable pH range for dermal preparations (the pH of the skin surface is between 4.2 and 5.6). The presence of wax led to an increase in pH, except in the case of laurel wax. No significant change in pH was observed when the wax concentration of the ointments was increased. Acidic pH values were expected due to the high concentration (40% *w*/*w*) of free FAs. FAs are weak acids with a pKa value of about 4.8 that increases slightly with the increasing chain length [29]. In general, it is recommended that the pKa value for a drug to be used is between 4 and 8 to avoid itching or skin irritation [30]. Irritation or redness at the site of application is frequently observed if the active ingredients have a pH different from that of the skin, especially in people with tender skin [31,32].

Besides the therapeutic efficacy, sensory acceptability also decides the marketing success of the topical formulation. Formulating a product that possess both optimal efficacy and expected sensory qualities can be extremely challenging [33]. The analysis of sensory profile is not a straightforward process, but frequently it is related to the physiological properties of the product ascribed by the formulation’s homogeneity, consistency or firmness [34]. Factors that can influence the homogeneity of the ointment include the solidification profile of the composition during its preparation such as the cooling rate, and variations in preparing techniques. Except for the type of wax used, all the ointment components were identical, as was the method of preparation.

### 3.3. Stability Studies

Fish oil is particularly susceptible to rancidity due to its high content of omega-3 PUFAs which are highly prone to oxidation. Monitoring the PV of the ointments allowed us to determine the fatty acid rancidification. As can be seen in Figure 1, no correlation between PV and presence of wax was found, and the increased wax concentration did not alter the PV of any of the ointments tested. In all cases, except in formulation O1, there was a rapid increase in the peroxide value during the first month of storage at 40 °C. A possible explanation could be the presence of oxygen in the headspace of the sealed tubes. After that, the PV increased only slightly with time under both ambient and accelerated conditions (*i.e.*, at 40 °C). It was evident that oxidation of the PUFAs occurred under both ambient and accelerated storage conditions, but it was independent of ointment composition. However, there was very little lipid peroxidation, as the PVs in all of the ointments were below the suggested limit for acceptability and quality for fish oils for human consumption. All formulations tested met the quality standards of the European Pharmacopeia [35].

Stability studies of ointments were further supported by the evaluation of sensory parameters (data not shown). All ointments containing microcrystalline wax, rice bran wax, laurel wax or beeswax were stable during the six months of storage at room temperature and displayed no change in texture, color or smell. A deviation from initial sensory parameters was observed in the case of C1, C3, O1, O2, Cd1 and Cd2 after six months of storage under ambient conditions as well as at 40 °C. A slight change in color in the formulations at 40 °C and “bleeding“ after 90 days was observed. The phenomenon of bleeding was attributed to the storage at high temperature which, in fact, is a well-known change in consistency that typically appears on the top of a filled tube and is frequently referred to as the main stability problem of ointments [36]. According to the results obtained from a short-duration stability study, C1, C2, O1, O2, Cd1, Cd2 and all formulations containing rice bran, laurel or beeswax were slightly different in homogeneity than the original samples. However, besides the textural and sensory changes, phase separation was not observed.

## 4. Materials and Methods

### 4.1. Antibacterial Assay

The strains selected to use for the antibacterial assay were *Staphylococcus aureus* (ATCC2913), *Enterococcus faecalis* (ATCC29212), *Streptococcus pyogenes* (ATCC19615) and *Streptococcus pneumoniae* (ATCC49169) representing common Gram-positive species, and *Pseudomonas aeruginosa* (ATCC27853), and *Escherichia coli* (ATCC 25922) representing Gram-negative species. MIC determinations were performed using agar dilution according to the CLSI standard for methods for dilution antimicrobial tests for bacteria that grow aerobically [37]. Double dilutions of FAs extract of cod liver oil were prepared. One mL of each included 381 μL of FAs, 200 μL ethanol and 419 μL of sterile distilled water that was added to 19 mL of melted Mueller-Hinton agar (Oxoid, Hampshire, UK), mixed and poured in sterile Petri dishes to obtain the final concentrations of 1–16.384 μg/mL. For the MIC testings of streptococci the agar was supplemented with 5% sheep blood. Bacterial suspensions were made to include approximately 10^7^ CFU/mL, to give the final number of 10^4^ CFU in the 1 μL that was placed on the agars that were incubated at 37 °C for 18–24 h. The MIC determinations were done two times for each strain and dilution. Various concentrations of ethanol (20%, 40%, 60%, 80%, and 100%) (Sigma Aldrich, Steinheim, Germany) without FAs extract was used to evaluate the effect of the ethanol. Mueller Hinton agar without alcohol and FAs extract was used for growth control.

### 4.2. Ointment Preparation

The fatty acid extract was supplied by Lýsi Ltd. (Reykjavik, Iceland).The method to determine the content and composition of FAs present in cod liver oil and its extract has been previously described [13]. Vaseline was purchased from Duchefa Farma (Haarlem, The Netherlands), carnauba wax, ozokerite wax, laurel wax, candelilla wax, rice bran wax were purchased from Strahl&Pitch (West Babylon, NY, USA), and beeswax and microcrystalline wax were purchased from Making cosmetics (Snoqualmie, WA, USA), coconut butter was purchased from Fagron (Newcastle upon Tyne, UK), lavender oil and lemon oil were purchased from Now Foods (Bloomingdale, IL, USA).

Twenty-one formulations were prepared by fusion method, in which the FAs extract (40% *w*/*w*) was combined with a melted wax base and evaluated for physicochemical parameters [38]. All the compositions are summarized in Table 2. Free FAs extract was heated gently on a water bath at 50 °C until the entire content has melted. Simultaneously, wax was completely melted under stirring. When wax base had melted, both Vaseline and coconut oil were added. Free FAs extract was added slowly to the mixture under gentle stirring. Then, lavender oil and lemon oil were added and mixed together with Vaseline, coconut oil and waxes. The ointments were filled in airtight aluminum tubes in order to prevent access of light and oxygen.

### 4.3. Evaluation of Ointments

#### 4.3.1. Spreadability

A simple spreadability test was performed: 1.0 g of the ointment was placed in a 10-mm-diameter circle on a glass plate. After being sandwiched with another glass plate, the sample was pressed with fixed 500 g weight [39]. Spreadability was determined as a difference in diameter values before and after 30 s on a millimeter scale placed under the lower glass plate.

#### 4.3.2. Extrudability

Tube extrudability was determined by measuring the amount of ointment extruded from the tube when a pressure was applied on the tube. The larger amount extruded the better extrudability. Extrudability was determined in terms of weight in grams required to extrude 0.5 cm of ribbon of ointment in 5 s [40].

#### 4.3.3. pH Determination

The pH of ointments were determined by an extraction method where 2.5 g of ointment was suspended in 50 mL distilled water in a 100 mL beaker, heated in a water bath at about 70 °C for 10 min, and then cooled to room temperature. The suspension was vigorously stirred for 10 min on magnetic stirrer to prompt extraction before phase separation resulting in supernatant extract [41]. The pH was measured using a PH-200 Waterproof pH Meter.

#### 4.3.4. Sensory Evaluation

Sensory parameters of ointments were evaluated at different storage conditions, that is 40 ± 2 °C, 75% RH ± 5% RH for three months and under ambient condition for six months. The colors, odors and textures of the ointments were evaluated and compared. A standardized quantity of 1 g of ointment was applied over the dorsal surface of the left hand and sensory properties descriptively graded using terms as follows:

(1) Excellent: smooth and homogeneous texture, light brown color, distinctive fishy odor.
(2) Satisfactory: smooth texture, non-consistency, light brown color, distinctive fishy odor.
(3) Unsatisfactory: rough texture, visible solid particles, non- consistency, dark brown color, rancid fishy odor.

#### 4.3.5. Determination of Peroxide Value

A modified method of Wheeler described in ISO 3960:2007 was used where lipid peroxide is reacted with potassium iodide (KI) to liberate iodine that is then titrated with a standard solution of sodium thiosulfate (Na_2_S_2_O_3_). The amount of liberated iodide represents the peroxide content. PV is defined as the amount of peroxides oxygen per 1 kg fat (mmol O_2_/kg). Aluminum tubes were kept at room temperature with no access of oxygen, during six months period. All the developed formulations were also subjected to accelerated stability at testing at 40 ± 2 °C, 75% RH ± 5% RH for three months. For three months accelerated study, PV was assessed at one, two and three months.

## 5. Conclusions

In the current trend for the development of natural and safe antibacterial compounds with minimal side effects, although not potent, fatty acid extract from fish liver oil has proved to be an effective antibacterial agent against Gram-positive bacteria. As such, fish oil holds a great potential as an active pharmaceutical ingredient for novel effective pharmaceutical formulations, especially for the prevention and treatment of skin diseases. The ointments were shown to have a great potential as an effective and safe way to administer PUFAs for topical antimicrobial therapy. In addition, it may be concluded that waxes could be used as suitable excipients for ointments creating compositions with appreciated rheological and sensory profiles that perform safe and overall effective dermal therapy.

## Figures and Tables

**Table 1:** Typical fatty acid composition of fatty acid extract from cod liver oil [13].

Fatty Acid	Amount (%)	
Name	Shorthand	
Saturated fatty acids		
Myristic acid	14:0	3.6
Palmitic acid	16:0	10.4
Stearic acid	18:0	2.6
Monounsaturated fatty acids		
Palmitoleic acid	16:1 *n*-7	6.5
*cis*-Vaccenic acid	18:1 *n*-7	4.4
Oleic acid	18:1 *n*-9	16.2
Gondoic acid	20:1 *n*-9	9.4
Gadoleic acid	20:1 *n*-11	1.6
Erucic acid	22:1 *n*-9	0.6
Cetoleic acid	22:1 *n*-11	7.8
Polyunsaturated fatty acids		
Linoleic acid	18:2 *n*-6	1.5
Moroctic acid	18:4 *n*-3	2.4
Eicosapentaenoic acid (EPA)	20:5 *n*-3	9.3
Docosahexaenoic acid (DHA)	22:6 *n*-3	11.9

**Table 2:** Composition (% *w*/*w*) of ointments.

Formulation Code	Compound (% *w*/*w*)					
	Fatty Acid Extract	Vaseline	Coconut Butter	Wax	Lavender Oil	Lemon Oil
C1, O1, L1, B1, Cd1, Rb1, M1	40	43.5	14	1	1	0.5
C2, O2, L2, B2, Cd1, Rb2, M2	40	43.5	12	3	1	0.5
C3, O3, L3, B3, Cd3, Rb3, M3	40	43.5	10	5	1	0.5

**Table 3:** Minimum inhibitory concentration (MIC) for the fatty acid extract.

Strains	*Staphylococcus aureus*	*Streptococcus pyogenes*	*Streptococcus pneumonia*	*Enterococcus faecalis*	*Pseudomonas aeruginosa*	*Escherichia coli*
	ATCC	ATCC	ATCC	ATCC	ATCC	ATCC
	29213	19615	49169	29212	27853	25922
MIC (μg/mL)	512	256	128	256	>16.384	>16.384

**Table 4:** Spreadability, extrudability and pH values of ointments.

Formulation	Spreadability (mm ± SD)	Tube Extrudability (g ± SD)	pH
C1	21.50 ± 0.87	3.23 ± 0.19	5.44 ± 0.04
C2	14.66 ± 0.20	1.84 ± 0.05	5.21 ± 0.01
C3	13.33 ± 0.57	1.67 ± 0.10	5.06 ± 0.05
O1	19.66 ± 0.57	3.23 ± 0.39	4.70 ± 0.03
O2	15.66 ± 0.2	2.84 ± 0.62	4.62 ± 0.07
O3	12.66 ± 1.5	1.69 ± 0.53	4.48 ± 0.02
L1	25.33 ± 2.51	2.50 ± 0.12	3.83 ± 0.03
L2	22.66 ± 0.57	2.15 ± 0.07	4.03 ± 0.005
L3	19.66 ± 1.52	2.04 ± 0.09	4.06 ± 0.05
B1	30.50 ± 1.32	4.84 ± 0.79	4.30 ± 0.03
B2	30.13 ± 0.28	3.95 ± 0.49	4.05 ± 0.05
B3	27.33 ± 0.57	3.32 ± 0.07	3.96 ± 0.01
Cd1	19.33 ± 1.15	3.30 ± 0.26	4.28 ± 0.04
Cd2	17.33 ± 0.57	3.20 ± 0.10	3.84 ± 0.02
Cd	17.66 ± 0.57	2.92 ± 0.36	3.83 ± 0.02
Rb1	22.50 ± 0.50	2.97 ± 0.67	4.85 ± 0.02
Rb2	18.66 ± 0.57	1.67 ± 0.34	4.54 ± 0.03
Rb3	17.33 ± 1.52	1.24 ± 0.16	4.32 ± 0.02
M1	24.66 ± 6.65	3.30 ± 0.26	4.24 ± 0.02
M2	21.66 ± 0.57	3.33 ± 0.14	4.21 ± 0.01
M3	18.66 ± 2.88	2.90 ± 0.13	4.09 ± 0.02

## Abbreviations

The following abbreviations are used in this manuscript:

FAs: Fatty acids
PUFAs: Polyunsaturated fatty acid
PV: Fatty acids
MIC: Minimum inhibitory concentration

## References

[1] Siriwardhana N., Kalupahana N.S., Moustaid-Moussa N. (2012). Health benefits of *n*-3 polyunsaturated fatty acids: Eicosapentaenoic acid and docosahexaenoic acid. Adv. Food Nutr. Res..

[2] Jump D.B., Depner C.M., Tripathy S. (2012). Omega-3 fatty acid supplementation and cardiovascular disease. J. Lipid Res..

[3] Shahidi F. (2006). Nutraceutical and Specialty Lipids and Their Co-Products.

[4] Sun C.Q., O’Connor C.J., Roberton A.M. (2003). Antibacterial actions of fatty acids and monoglycerides against Helicobacter pylori. FEMS Immunol. Med. Microbiol..

[5] Dilika F., Bremner P.D., Meyer M.J.J. (2000). Antibacterial activity of linoleic and oleic acids isolated from Helichrysum pedunculatum: A plant used during circumcision rites. Fitoterapia.

[6] Thormar H., Issacs C.E., Brown H.R., Marc R., Barshatzky M.R., Pessolono T. (1987). Inactivation of Enveloped Viruses and Killing of Cells by Fatty Acids and Monoglycerides. Antimcrob. Agents Chemother..

[7] Thibane V.S., Kock J.L., Ells R., Wyk P.W., Pohl C.H. (2010). Effect of marine polyunsaturated fatty acids on biofilm formation of *Candida albicans* and *Candida dubliniensis*. Mar. Drugs.

[8] Walters D., Raynor L., Mitchell A., Walker R., Walker K. (2004). Antifungal activities of four fatty acids against plant pathogenic fungi. Mycopathologia.

[9] Kabara J.J., Swieczkowski D.M., Conley A.J., Truant T.J.P. (1972). Fatty Acids and Derivatives as Antimicrobial Agents. Antimicrob. Agents Chemother..

[10] Loftsson T., Thormar H., Hjalmarsdottir M. (1997). Antibacterial activities of fatty acids extracts from cod liver oil.

[11] Loftsson T., Ilievska B., Asgrimsdottir G.M., Ormarsson O.T., Stefansson E. (2016). Fatty acids from marine lipids: Biological activity, formulation and stability. J. Drug Deliv. Sci. Technol..

[12] Loftsson T., Thormar H., Ólafsson J.H., Gunarsdottir T.M., Hjaltason B., Gudmundsson G. (1998). Fatty Acid Extract From Cod-liver Oil: Activity Against Herpes Simplex Virus and Enhancement of Transdermal Delivery of Acyclovir *In-vitro*. Pharm. Pharmacol. Commun..

[13] Loftsson T., Gudmundsdóttir T.K., Fridriksdóttir H., Sigurdardóttir A.M., Thorkelsson J., Gudmundsson G., Hjaltason B. (1995). Fatty acids from cod-liver oil as skin penetration enhancers. Pharmazie.

[14] Thormar H. (2011). Lipids and Essential Oils as Antimicrobial Agents.

[15] Desbois A.P., Smith V.J. (2009). Antibacterial free fatty acids: Activities, mechanisms of action and biotechnological potential. Appl. Microbiol. Biotechnol..

[16] Ueda C.T., Shah V.P., Derdzinski K., Ewing G., Flynn G., Maibach H., Marques M., Rytting H., Shaw S., Thakker K. (2009). Topical and Transdermal Drug Products. Pharmacop. Forum.

[17] Allen L., Ansel C.H. (2013). Ansel’s Pharmaceutical Dosage Forms and Drug Delivery Systems.

[18] Harwood J.L., Gurr M.I. (1991). Lipid Biochemistry: An Introduction.

[19] Budai L., Antal I., Klebovich I., Budai M. (2012). Natural oils and waxes. J. Cosmet. Sci..

[20] Darvell B.W. (2009). Materials Science for Dentistry.

[21] Nasopoulou C., Karantonis H.C., Andriotis M., Demopoulos C.A., Zabetakis I. (2008). Antibacterial and anti-PAF activity of lipid extracts from sea bass (*Dicentrarchus labrax*) and gilthead sea bream (*Sparus aurata*). Food Chem..

[22] McGaw L.J., Jager A.K., Van Staden J., Houghton P.J. (2002). Antibacterial effects of fatty acids and related compounds from plants. South Afr. Bot..

[23] Chaudhari S.P., Patil P.S. (2012). Pharmaceutical Excipients: A review. Int. J. Adv. Pharm. Biol. Chem..

[24] Chang R.K., Raw A., Lionberger R., Yu L. (2013). Generic development of topical dermatologic products: Formulation development, process development, and testing of topical dermatologic products. AAPS J..

[25] Kryscio D.R., Sathe P.M., Lionberger R., Yu L., Bell M.A., Jay M., Hilt J.Z. (2008). Spreadability measurements to assess structural equivalence (Q3) of topical formulations—A technical note. AAPS PharmSciTech.

[26] Ivens U.I., Steinkjer B., Serup J., Tetens V. (2001). Ointment is evenly spread on the skin, in contrast to creams and solutions. Br. J. Dermatol..

[27] Noren B. (1976). A method to evaluate the tubesqueezing properties of toothpaste. J. Soc. Cosmet. Chem..

[28] Shellhammer T.H., Rumsey T.R., Krochta J.M. (1997). Viscoelastic properties of edible lipids. J. Food Eng..

[29] Kanicky J.R., Shah D.O. (2002). Effect of Degree, Type, and Position of Unsaturation on the pKa of Long-Chain Fatty Acids. J. Colloid Interface Sci..

[30] Paudel S.K., Milewski M., Swadley L.C., Brogden K.N., Ghosh P., Stinchcomb A.L. (2010). Challenges and opportunities in dermal/transdermal delivery. Ther. Deliv..

[31] Nangia A., Andersen P.H., Berner B., Maibach H.I. (1996). High dissociation constants (pKa) of basic permeants are associated with *in vivo* skin irritation in man. Contact Dermat..

[32] Ali S.M., Yosipovitch G. (2013). Skin pH: From basic science to basic skin care. Acta Derm. Venereol..

[33] Almeida I.F., Gaio A.R., Bahia M.F. (2008). Hedonic and descriptive skin feel analysis of two oleogels: Comparison with other topical formulations. J. Sens. Stud..

[34] Hootman R.C. (1992). Manual on Descriptive Analysis Testing for Sensory Evaluation.

[35] EFSA Panel on Biological Hazards (BIOHAZ) (2010). Scientific Opinion on Fish Oil for Human Consumption. Food Hygiene, including Rancidity. EFSA J..

[36] Loyd V.A. (2013). Remington: An Introduction to Pharmacy.

[37] (2009). Methods for Dilution Antimicrobial Susceptibility Tests for Bacteria That Grow Aerobically; Approved Standard—Eighth Edition.

[38] Gad C.S. (2008). Pharmaceutical Manufacturing Handbook: Production and Processes.

[39] Contreras M.D., Sanchez R. (2002). Application of a factorial design to the study of the flow behavior, spreadability and transparency of a Carbopol ETD 2020 gel. Part II. Int. J. Pharm..

[40] Viswanad V., Aleykutty N.A., Jayakar B., Zacharia S.M., Thomas L. (2012). Development and evaluation of antimicrobial herbal formulations containing the methanolic extract of Samadera indica for skin diseases. J. Adv. Pharm. Technol. Res..

[41] Pasupathi A., Palanisamy P.B., Jaykar R., Chandira M., Venkateswarlu B.S. (2009). Formualtion, development, evaluation of calcitriol and clobetasol propionate ointment. Indian J. Res. Pharm. Biotechnol..

